# Seasonal differences and potential biological drivers of the methane paradox in two peri-Alpine lakes

**DOI:** 10.1002/lol2.70129

**Published:** 2026-04-30

**Authors:** Niharika Sharma, Manuela Felsberger, Zeynep Kurt, Markus Möst, Barbara Bayer

**Affiliations:** 1 Division of Microbial Ecology, Centre for Microbiology and Environmental Systems Science, University of Vienna, Vienna, Austria; 2 Research Department for Limnology, University of Innsbruck, Innsbruck, Austria

## Abstract

Seasonal variations and the biological drivers underlying the methane paradox in freshwater lakes are poorly understood. Here, we investigated the relationship between subsurface methane inventories and phytoplankton in two peri-Alpine lakes across different seasons. Surface waters of both lakes were consistently saturated in methane, with maxima reaching 570 and 205 nmol L^−1^ during summer in the metalimnion of lakes Mondsee and Attersee, respectively. Methane concentrations were positively correlated with phytoplankton abundance in meso-oligotrophic lake Mondsee but not in ultra-oligotrophic lake Attersee. However, although phytoplankton peaked in abundance at the methane maxima in Mondsee, incubation experiments with ^13^C-labeled bicarbonate revealed negligible methane production from primary productivity. Instead, our results suggest that phytoplankton might only be indirectly involved in methane production through alternative pathways, or by providing precursor compounds to other members of the microbial community in these oligotrophic lakes.

Methane (CH_4_) is the second most important greenhouse gas after carbon dioxide, contributing approximately 25% to global warming since the industrial revolution (Ganesan et al. 2019; IPCC 2023). Freshwater ecosystems emit the largest amount of CH_4_, with lakes alone estimated to emit 151 ± 73 Tg CH_4_ per year (Rosentreter et al. 2021). Traditionally, CH_4_ production in aquatic ecosystems has been attributed to methanogenesis in anoxic sediments (Bastviken 2009). However, this view has been challenged by increasing evidence for CH_4_ production in oxygenated waters of both oceans and freshwater lakes (Karl et al. 2008; Grossart et al. 2011; Schroll et al. 2023; Mao et al. 2024). The occurrence of elevated CH_4_ concentrations in oxic surface waters is referred to as the methane paradox and might account for 18–90% of total CH_4_ emissions from freshwater lakes to the atmosphere (Donis et al. 2017; Günthel et al. 2020). Growing evidence suggests a link between elevated CH_4_ concentrations ([CH_4_]) and subsurface chlorophyll (Chl) maxima (Bogard et al. 2014; Tang et al. 2014; Donis et al. 2017; Günthel et al. 2020; Thottathil et al. 2022; Ordóñez et al. 2023), indicating a contribution of phytoplankton activity to the methane paradox. A relationship between phytoplankton and CH_4_ production has also been demonstrated in both culture experiments (Lenhart et al. 2016; Klintzsch et al. 2019; Bižić et al. 2020) and field-based studies using stable isotope tracing approaches (Tang et al. 2014; Günthel et al. 2020; Morana et al. 2020). However, while a direct link to photosynthesis has been proposed previously (Bižić et al. 2020; Günthel et al. 2020), the exact mechanisms involved in CH_4_ production by phytoplankton remain unresolved.

Phytoplankton blooms are expected to become more frequent during future climate scenarios (Jones and Brett 2014), which could lead to higher CH_4_ emissions from lake ecosystems (Beaulieu et al. 2019). Consequently, understanding the role of phytoplankton in seasonal CH_4_ dynamics is essential to better understand and predict future CH_4_ emission scenarios. Furthermore, despite its widespread occurrence, the methane paradox is not a universal phenomenon and may be absent in some lakes (Khatun et al. 2019; Ordóñez et al. 2023). This highlights the importance of understanding the influence of environmental and seasonal dynamics on the occurrence of the methane paradox in lake ecosystems.

In this study, we investigated seasonal CH_4_ dynamics in oxic surface waters of the two Austrian lakes Mondsee and Attersee, which are located in the Alpine foothills and formed by tectonic-glacial processes (= peri-Alpine; Tolotti et al. 2018). Both lakes lie within the same catchment but differ in trophic state, offering a natural contrast for comparison. Our primary objective was to understand the biological drivers, particularly the role of phytoplankton, in the formation of the methane paradox in these lakes.

## Methods

### Study site and sampling procedures

**Fig. 1:**
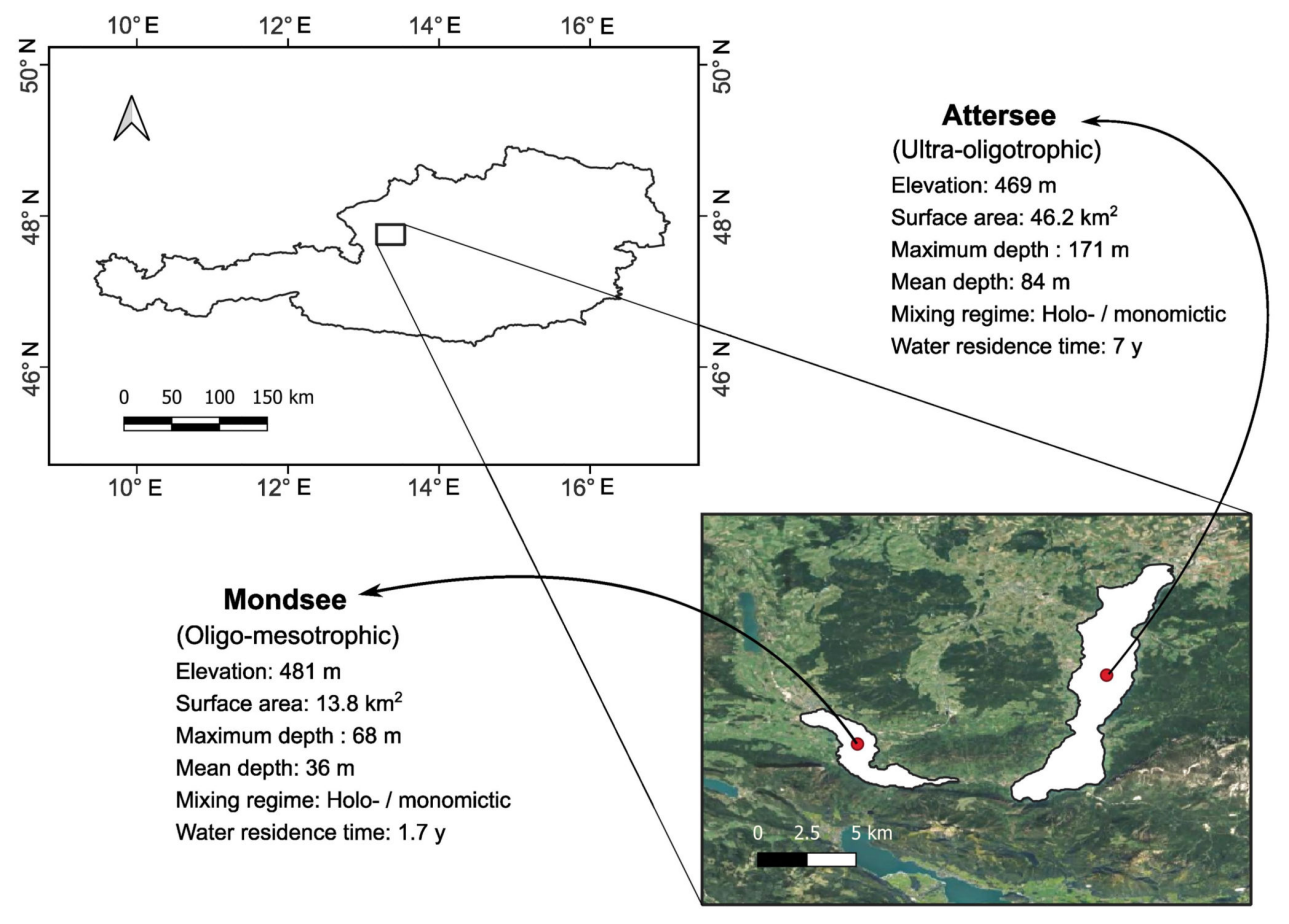
Study area. Morphometric data is taken from the annual report on ecological state of the lakes in the province of Upper Austria (Krisa and Hainz 2024).

The study was conducted in a twin lake system, Mondsee and Attersee, located in the Salzkammergut region of Austria (Fig. 1). Attersee is a deep ultra-oligotrophic lake while Mondsee is an oligo-mesotrophic lake. Water samples were collected from both lakes at their deepest points during 17–18 July 2024 (summer), 5–6 November 2024 (autumn), 1–2 April 2025 (spring), and 24–25 June 2025 (summer). Water column profiles of temperature, dissolved oxygen (DO), Chl, phycoerythrin (PE), and conductivity were recorded with a multiparameter probe (YSI 6600 V2, Ohio, USA). PE is an accessory pigment present in some cyanobacteria and eukaryotic phytoplankton (Bryant 1982). Despite prior calibration of fluorescence probes with Milli-Q, some profiles in lake Attersee showed continuously negative values, possibly as a result of the low pigment abundance in this ultra-oligotrophic lake. Consequently, we additionally collected discrete water samples for Chl *a* analysis from both lakes in the latter two sampling events. In situ light measurements were taken using a LI-COR light sensor (LI-193, LiCOR, Lincoln, USA) whenever sampling was performed during midday and cloud coverage was minimal. Water samples were collected with a 10 L Niskin bottle, targeting discrete depths within and below the euphotic zone, ranging from 0 to 40 m in Mondsee and from 0 to 60 m depth in Attersee.

### Methane concentration analysis

Samples for dissolved CH_4_ concentrations were collected directly from the Niskin bottle using silicon tubing (Masterflex, Germany). Duplicate water samples were collected in 120 mL glass serum vials filling from bottom and overflowing 2 to 3 times to remove air, and bottles were closed bubble-free with gray butyl rubber septa (#27232, Merck) and aluminum crimps. The rubber septa were pretreated by autoclaving twice with Milli-Q to prevent leaching from the septa. After preserving with 150 *μ*L saturated ZnCl_2_ solution, samples were stored in the dark at 14°C prior to analysis on a Picarro G2201-i analyzer (Picarro Inc., CA, USA). Information on instrument calibration and measurement procedures can be found in Supporting Information Text S1.

### Nutrient and chlorophyll analysis

Samples for ammonium (NH_4_^+^) and nitrite (NO_2_^−^) concentrations were collected directly from the Niskin bottle and analyzed on the same day. Samples for nitrate (NO_3_^−^) and phosphate (PO_4_^3−^) concentrations were collected after syringe-filtration (0.22 *μ*m, Ministar, Sartorius) and frozen at −20°C for later analysis. Samples for Chl *a* were collected in 1 L amber bottles and stored at 4°C in the dark until sample processing on the same day. Concentrations of NO_2_^−^, NO_3_^−^, and PO_4_^3−^ were measured colorimetrically, while that of NH_4_^+^ and Chl *a* were measured fluorometrically. Detailed methodological procedures for nutrient analysis are provided in Supporting Information Text S2.

### Cell abundance measurements

Samples for flow cytometry analysis were collected in 1.8 mL cryotubes (Carl Roth, Germany), fixed with glutaraldehyde (final concentration 0.5%) for 10 min, flash frozen in liquid nitrogen, and stored at −80°C (Marie et al. 1997) until analysis on a CytoFLEX S flow cytometer (Beckman Coulter, CA, USA). Prior to sample analysis, internal fluorescence calibration was performed with 3 *μ*m fluorescent microsphere beads (Beckman Coulter, CA, USA). A threshold of 750 was set to side scatter to optimize event and abort rate of the measurement, and the samples were analyzed at a flow rate of 30 *μ*L min^−1^. All data were collected on a log scale and analyzed using the inbuilt CytExpert Software (Beckman Coulter, CA, USA). Total cell abundance was quantified after staining with SYBR green I dye (1:10,000 dilution; Lonza Biosciences, Switzerland) for 10 min in the dark (Marie et al. 1997). Phyto-plankton cells were quantified by their autofluorescence (red fluorescence from Chl *a* and yellow fluorescence from PE) and scattering properties (side scatter and forward scatter) in unstained samples. Non-photosynthetic cells were identified by the difference between SYBR green positive cells and phytoplankton cells.

### Microbial community composition

Water was collected in acid-washed 2 L polycarbonate bottles and the biomass was sequentially filtered onto 3 *μ*m (25 mm, Isopore, Millipore) and 0.22 *μ*m (25 mm, Durapore, Millipore) poresize membrane filters, which were subsequently frozen in 2 mL gasketed bead-beating tubes (Lysis Matrix E, Qiagen) at −80°C until extraction. DNA was extracted using the Qiagen PowerSoil Pro kit as described in Supporting Information Text S3. The V4 hypervariable region of the bacterial and archaeal 16S rRNA gene was amplified using the V4-EXT primer pair (forward/reverse: GTGYCAGMMGBNKCGGTVA/RGACTAMNVRGGTHTCTAAT) (Hu et al. 2025). DNA extraction, sequencing, and raw data processing was performed at the Joint Microbiome Facility of the Medical University of Vienna and the University of Vienna following a standardized 2-step PCR protocol described previously (Pjevac et al. 2021), and sequenced on an Illumina MiSeq (2 × 300 bp) sequencer. Information on sequence data processing is provided in Supporting Information Text S3.

### Primary production associated methane production

CH_4_ production associated with primary production (= dissolved inorganic carbon [DIC] fixation) was assessed during summer 2024 and autumn 2024 to test the role/contribution of phytoplankton primary production to oxic CH_4_ formation. We collected water samples from the depths corresponding to Chl and PE fluorescence maxima as phytoplankton biomass is expected to be high at these depths. Water samples were collected as described above (*see* section on Methane concentrations) and ^13^C-labeled bicarbonate (98 atom%, Merck KGaA, Darmstadt, Germany) was added to incubation bottles to achieve a labeling of 10–20%. Samples were incubated for a maximum of 96 h at close to in situ temperature and light conditions (Supporting Information Table S1), and incubations were terminated by adding 150 *μ*L saturated ZnCl_2_ solution. CH_4_ concentrations and isotopic enrichment were measured with a Picarro G2201-i analyzer (Picarro Inc., CA, USA), following the methodological details described in Supporting Information Text S1. Separate water samples were collected to quantify in situ DIC concentrations (Supporting Information Text S1). Methane production rates were calculated from the increase of ^13^CH_4_ concentrations over time (Hama et al. 1983). Rates were considered significant when the slope of the linear regression was statistically different from zero (one-sided Student’s *t*-test, *p* < 0.05).

### Statistical analyses

Pearson correlation coefficients were calculated to quantify the strength and direction of relationships between [CH_4_] and environmental parameters for lakes and within different lake layers (epilimnion, metalimnion and hypolimnion) in seasons where a distinct metalimnion was identified. Subsequently, linear regression analysis was performed to describe the linear relationships of some of the observed correlations.

Metalimnion depth was calculated using a temperature gradient threshold of ≥ 1°C m^−1^. The shallowest and deepest temperature change exceeding this threshold was used to define the depth range of the metalimnion. For duplicate CH_4_ samples, the arithmetic mean was used for calculations. Statistical analysis and data visualization was conducted using R software (version 4.3.3) and Sigmaplot (version 14.0).

## Results

### Environmental context

**Fig. 2:**
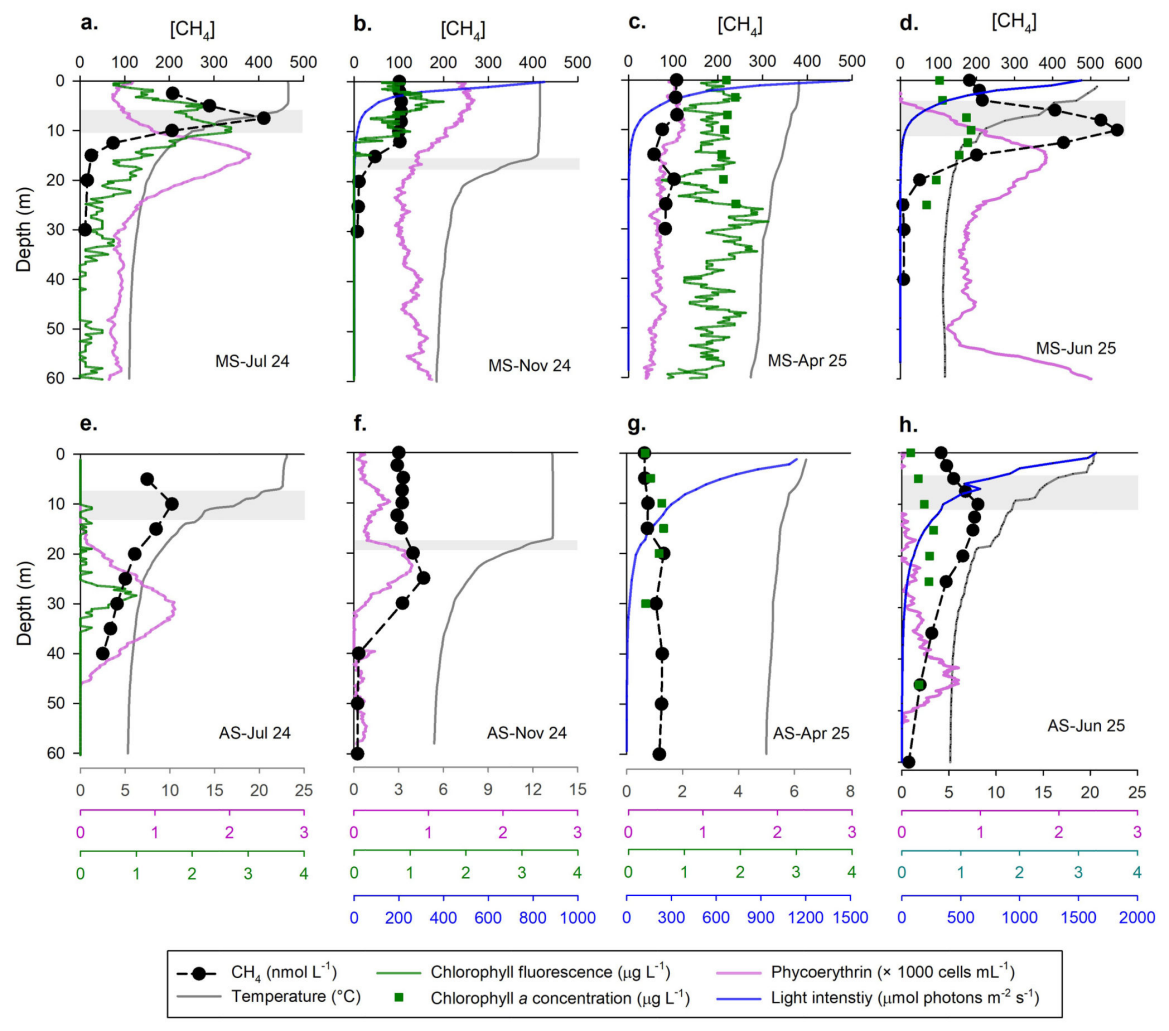
Vertical profiles of dissolved methane (CH_4_), temperature, chlorophyll, phycoerythrin, and light intensity in lakes Mondsee (MS, **a–d**) and Attersee (AS, **e–h**) during different seasons. Sampling time corresponding to each plot is denoted by month-year. Chlorophyll and phycoerythrin multiprobe profiles are not reported in some seasons due to continuously negative values (*see* Methods section). Sporadic negative fluorescence values were set to zero for the purpose of visualization. Light intensity was only measured during midday on clear, sunny days. The metalimnion is indicated by gray shaded boxes.

Average surface water temperature of lakes Mondsee and Attersee ranged from 5 to 23°C during different sampling events. Both lakes were thermally stratified with a distinct formation of epilimnion, metalimnion, and hypolimnion during summer and autumn (Fig. 2). In contrast, the water column was completely mixed in spring, indicating the persistent effects of winter turn-over. Irrespective of the season, the entire water column of both lakes remained oxic, ranging from 49 to 363 *μ*mol L^−1^ in Mondsee and from 268 to 353 *μ*mol L^−1^ in Attersee (Supporting Information Fig. S1, Sharma et al. 2026). The euphotic zone (defined as 0.5% of surface light levels; Wu et al. 2021), extended to approximately 16 m in Mondsee and 37 m in Attersee. Chl and PE fluorescence was consistently higher in Mondsee compared to Attersee (Fig. 2), corresponding to differences in trophic state between the two lakes. NH_4_^+^ and NO_2_^−^ reached maximum concentrations of 899 and 408 nmol L^−1^ in lake Mondsee and 640 and 333 nmol L^−1^ in lake Attersee, respectively, while NO_3_^−^ concentrations ranged from 15 to 47 *μ*mol L^−1^ in both lakes throughout the year (Supporting Information Fig. S1). Both lakes were phosphorus-limited, with PO_4_^3−^ concentrations below the detection limit (≤ 0.25 *μ*mol L^−1^) in most depth layers, corresponding to nitrogen: phosphorus (N : P) ratios of ≥ 52 : 1 throughout the year (Supporting Information Fig. S1).

Phytoplankton abundance ranged from 1.47 × 10^4^ to 2.46 × 10^5^ cells mL^−1^ in Mondsee and from 1.56 × 10^4^ to 2.52 × 10^5^ cells mL^−1^ in Attersee, whereas abundances of non-photosynthetic cells ranged from 8.58 × 10^5^ to 3.15 × 10^6^ cells mL^−1^ in Mondsee and from 6.87 × 10^5^ to 1.71 × 10^6^ cells mL^−1^ in Attersee (Supporting Information Fig. 2). Phytoplankton abundance peaked at the lower boundary of the metalimnion in Mondsee and below the metalimnion in Attersee, while non-photosynthetic cells were rather uniformly distributed in the epi- and metalimnion of both lakes (Supporting Information Fig. S2). Phytoplankton made up 1–7% and 2–15% of microbial cells in lakes Mondsee and Attersee, respectively, and mostly consisted of cells less than 3 *μ*m in size when compared to standard size beads (Supporting Information Fig. S3). Cyanobacteria made up the majority of phytoplankton during the summer season in both lakes in the 0.2–3 *μ*m size fraction, and almost exclusively consisted of members of the order Synechococcales (Supporting Information Fig. S4). In contrast, eukaryotic phytoplankton became relatively more abundant in autumn and spring in both lakes. Diatoms (Bacillariophyta) were the most abundant eukaryotic phytoplankton classified by chloroplast 16S rRNA gene sequences, with the exception of lake Mondsee in autumn, where *Cryptomonadaceae* made up most of the phytoplankton community (Supporting Information Fig. S4).

### Seasonal methane dynamics

The epilimnion of both lakes was consistently oversaturated with CH_4_ compared to atmospheric equilibrium (3 nM; Mao et al. 2024) (Fig. 2), with slightly higher concentrations in Mondsee (100–290 nmol L^−1^) than Attersee (96–149 nmol L^−1^). Depth-integrated CH_4_ concentrations were similar between both lakes and varied between 1.7–6.4 *μ*mol m^−2^ in Mondsee (0–30 m depth) and 2.5–4.8 *μ*mol m^−2^ in Attersee (0–40 m depth) throughout the year. [CH_4_] peaked within the metalimnion in summer in both lakes, with maximum values in Mondsee in June 2025 (570 nmol L^−1^) (Fig. 2). In autumn, deepening of the surface mixed layer led to uniform concentration within the upper 15 m of both lakes, while [CH_4_] peaked just below the metalimnion to 155 nmol L^−1^ in Attersee. No clear vertical pattern in [CH_4_] distribution was observed in spring in either lake as a result of winter mixing.

**Fig. 3:**
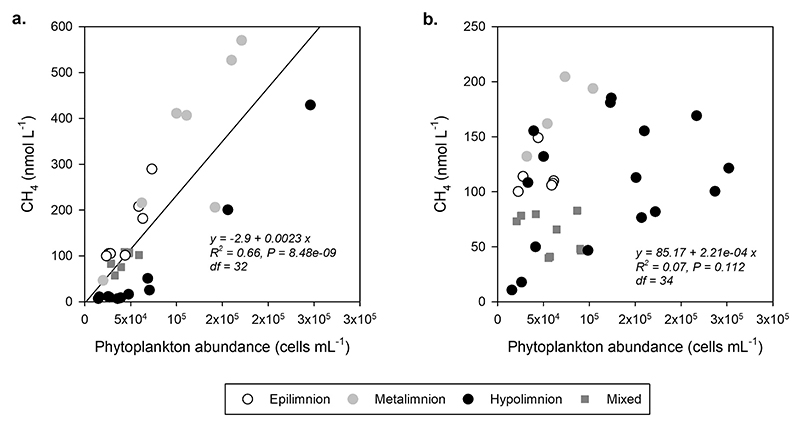
Relationship between methane concentrations and phytoplankton abundance in lakes (**a**) Mondsee and (**b**) Attersee. Samples corresponding to different lake layers (epilimnion, metalimnion and hypolimnion) are indicated by different colors. Samples from April, where no distinct metalimnion could be identified (indicative for a mixed water column), are shown by square shapes. Linear regression analysis is shown for all samples from lake Mondsee (*n* = 39) and lake Attersee (*n* = 42).

We observed a statistically significant linear relationship between [CH_4_] and phytoplankton abundance in lake Mondsee (*R*^2^ = 0.66, Fig. 3a), which was consistent across lake layers (epilimnion: *R*^2^ = 0.82, *p* = 0.0051; metalimnion: *R*^2^ = 0.67, *p* = 0.0241; hypolimnion: *R*^2^ = 0.95, *p* < 0.0001).

In contrast, no statistically significant relationship between phytoplankton and [CH_4_] was observed in lake Attersee (*R*^2^ = 0.07, Fig. 3b). Lake level correlation analysis further indicated a positive association between non-photosynthetic cells and [CH_4_] in the meta- and hypolimnion of lake Mondsee (Supporting Information Fig. S5), which was absent in lake Attersee (Supporting Information Fig. S6). No consistent positive correlation between [CH_4_] and nutrient concentrations was observed (Supporting Information Figs. S5 and S6), yet, [CH_4_] profiles closely resembled NH_4_^+^ concentration profiles in both lakes in autumn and in Mondsee in summer 2024. To further explore links between primary production and CH_4_ production, we performed stable isotope tracing experiments with ^13^C-bicarbonate in summer and autumn 2024 at the depths of the Chl and PE maxima. However, no increase in ^13^C-CH_4_ concentrations was observed during the incubation period (24–96 h) across all tested depths and seasons in both lakes (Table 1).

## Discussion

We investigated seasonal CH_4_ dynamics in the water columns of the two Austrian peri-Alpine lakes Mondsee and Attersee. In agreement with previous observations in temperate lakes (Bastviken 2009; Tang et al. 2016; Bartosiewicz et al. 2023), we identified a pronounced methane paradox in both lakes, with highest [CH_4_] within the metalimnion, coinciding with seasonal stratification during summer (Fig. 2). Though concentrations were highest in summer, [CH_4_] was elevated throughout the water columns of both lakes during all sampling events, indicating year-round CH_4_ over-saturation. These findings are in stark contrast to previous studies which reported clear seasonal differences, with CH_4_ often disappearing in winter (Schulz et al. 2001; Khatun et al. 2019). Our results together with previous observations suggest a high variability in seasonal CH_4_ dynamics between different lakes (Tang et al. 2014; Khatun et al. 2019; Günthel et al. 2020; Liu et al. 2024), yet distinctions between continuous and episodic CH_4_ production cannot be made with our current dataset.

Both lakes remained oxic throughout the year, limiting the possibility for the formation of low-oxygen niches to support canonical methanogenesis pathways (Bastviken et al. 2008). In support of this observation, 16S rRNA gene sequences of methanogenic archaea were not detected in the water columns of both lakes across all seasons (Sharma et al. 2026). The transport of CH_4_ from the littoral sediment zone as a potential contributor to sub-surface CH_4_ accumulation (Murase et al. 2005) also seems unlikely in these deep, oxic lakes, where CH_4_ oversaturation in surface waters is largely decoupled from sedimentary sources (Günthel et al. 2019).

We further explored the role of phytoplankton in the formation of the methane paradox in lakes Mondsee and Attersee. Despite similar phytoplankton composition in both lakes, a significant positive linear relationship between [CH_4_] and phytoplankton abundance was only observed in meso-oligotrophic lake Mondsee and not in ultra-oligotrophic lake Attersee (Fig. 3), suggesting that lake trophic state might influence phytoplankton-associated CH_4_ production. Our study does not take into account potential CH_4_ consumption, which might also shape [CH_4_] profiles. Methanotrophic bacteria were present throughout the water columns of both lakes in every season, making up between 0.1–2.1% and 0.4–2.3% of the microbial community in Mondsee and Attersee, respectively (Sharma et al. 2026). However, while CH_4_ consumption could potentially explain the lack of a relationship between [CH_4_] and phytoplankton abundance in lake Attersee, in situ CH_4_ oxidation rate measurements are required to derive any conclusions.

Cyanobacterial Synechococcales and diatoms were particularly abundant in both lakes throughout most of the year (Supporting Information Fig. S4) and have previously been suggested to be associated with the seasonal formation of CH_4_ maxima in freshwater lakes (Khatun et al. 2019; Günthel et al. 2020). However, contrary to previous studies which reported substantial CH_4_ production from primary production (0.2–11 nM d^−1^; Günthel et al. 2020; Morana et al. 2020), we observed no significant production of ^13^CH_4_ from ^13^C-bicarbonate in lakes Mondsee and Attersee (Table 1). Consequently, DIC fixation likely only plays a very minor role in the formation of the methane paradox in both lakes. However, phytoplankton could be involved in the production of CH_4_ through pathways other than DIC fixation. CH_4_ might be released during the breakdown of methylated substrates including methylphosphonate (MPn) and methylamine (Karl et al. 2008; Bižić-Ionescu et al. 2019; Wang et al. 2021). Both lakes were strongly P-limited, indicating that microbes might satisfy their cellular P demand by demethylating organic phosphonate compounds such as MPn (Karl et al. 2008; von Arx et al. 2023). Some members of freshwater Synechococcales encode the phosphonate degradation gene cluster (*phn* operon) (Zhao et al. 2022), suggesting the potential to use MPn as alternative P source. Alternatively, phytoplankton might also release organic compounds, which could be further broken down by heterotrophic microbes thereby releasing CH_4_ (Repeta et al. 2016; Wang et al. 2021). The dependence of CH_4_ production on in situ algal dissolved organic carbon release has been demonstrated previously (Bogard et al. 2014), and a positive correlation between non-photosynthetic cell abundance and dissolved CH_4_ in the meta- and hypolimnion of lake Mondsee (Supporting Information Fig. S5) could be indicative of such a coupling.

Taken together, our results suggest that despite differences in their trophic state, both lakes Mondsee and Attersee are a source of CH_4_ to the atmosphere throughout the year. While phytoplankton might play a role in regulating CH_4_ dynamics in oxic surface water of meso-oligotrophic lake Mondsee, CH_4_ production does not seem to be directly associated with primary production in these peri-Alpine lakes. Our results emphasize the need for more in-depth studies to resolve oxic CH_4_ production pathways and sub-seasonal differences in CH_4_ dynamics to predict CH_4_ emissions from freshwater lakes, particularly in lakes at higher elevation, where climatic changes occur at faster rates compared to the global average (Thompson et al. 2005; Råman Vinnå et al. 2021).

## Supplementary Material

Additional Supporting Information may be found in the online version of this article.

Supporting_Information

## Figures and Tables

**Table 1:** Primary productivity-associated methane production determined via ^13^C-bicarbonate stable isotope tracing incubation experiments in lakes Mondsee and Attersee. The change in ^13^CH_4_ concentration (average ± standard deviation) during the incubation period from the start of the incubation time (*T*_0_) until the end of the incubation time after 24 to 96 h (*T*_2_) are shown. ^13^CH_4_ concentrations were calculated from total CH_4_ concentrations and isotopic values (d^13^C-CH_4_) (Sharma et al. 2026). Incubation times varied between experiments (details are provided in Supporting Information Table S1). Sampling depth denotes the lake depth where water for incubation experiments was taken from, roughly corresponding to pigment maxima (*see* Methods section).

Lake	Season	Sampling depth (m)	^13^CH_4_ (nmol L^−1^)		
			*T*_0_	T_1_	T_2_
Mondsee	Summer	7.5	2.36 ± 0.18	2.41 ± 0.03	2.25 ± 0.29
Mondsee	Summer	15	0.20 ± 0.01	0.16 ± 0.00	0.18 ± 0.01
Mondsee	Autumn	4	0.67 ± 0.11	0.76 ± 0.02	0.72 ± 0.02
Mondsee	Autumn	10	0.84 ± 0.01	0.76 ± 0.01	0.68 ± 0.03
Attersee	Summer	10	n.a^*^	0.97 ± 0.01	0.98 ± 0.01
Attersee	Summer	25	0.52 ± 0.01	0.52 ± 0.01	0.53 ± 0.02
Attersee	Autumn	10	0.78 ± 0.00	0.77 ± 0.00	0.74 ± 0.04
Attersee	Autumn	20	1.09 ± 0.01	1.09 ± 0.02	0.99 ± 0.01

* Samples are not available (n.a.) due to human error.

## Data Availability

Data supporting the findings of this study are available in the Zenodo repository (https://doi.org/10.5281/zenodo.19394022). Raw 16S rRNA gene amplicon sequencing data is available at the Sequence Read Archive under the Bioproject Accession PRJNA1422683.

## Scientific Significance Statement

Methane oversaturation in oxygenated surface waters (= “methane paradox”) is a commonly observed phenomenon in fresh-water lakes and might contribute substantially to methane emissions due to its proximity to the atmosphere. Understanding the underlying mechanisms involved in the formation of the methane paradox is therefore essential to better predict future climate scenarios. In this study, we investigated seasonal methane dynamics in oxic surface waters of two Austrian peri-Alpine lakes which differ in their trophic state. We show that primary production is not directly linked to the formation of the methane paradox in both lakes. Our results further indicate that irrespective of their trophic state, peri-Alpine lakes might be a significant source of methane throughout the year.
