# Supplementary material for: Seasonal differences and potential biological drivers of the methane paradox in two peri-Alpine lakes
Source: Limnol Oceanogr Lett. Author manuscript; Available in PMC 2026 Jun 18. (PMC7619199; doi:10.1002/lol2.70129)
Supplement: Supporting_Information [file EMS214247-supplement-Supporting_Information.pdf]

Supporting Information

for

Seasonal differences and potential biological drivers of the methane paradox in two peri-Alpine lakes

Niharika Sharma[1], Manuela Felsberger[1], Zeynep Kurt[1], Markus Möst[2], and Barbara Bayer[1*]

[1]Division of Microbial Ecology, Centre for Microbiology and Environmental System Science, University of Vienna, 1030 Vienna, Austria

[2]University of Innsbruck, Research Department for Limnology, Mondsee, 5310 Mondsee/6020 Innsbruck, Austria

*Correspondence: Barbara Bayer, barbara.bayer@univie.ac.at

Contents of this file

Texts S1 to S3

Table S1

Figures S1 to S6

References

Text S1. Dissolved methane and inorganic carbon concentration measurements

The concentrations of dissolved methane [CH_4] were analyzed in samples preserved with $ZnCl_2$ following headspace equilibration method within 10 days of sample collection. For the analysis of [CH_4], 60 mL water samples were transferred from glass serum bottles into a 140 mL polypropylene fixed luer-lock syringe (Dissolution Accessories), and a 60 mL headspace was created using methane-free synthetic air (Roberts and Shiller 2015). After vigorously shaking for 1 minute, the headspace was diluted with synthetic air, and a total of 120 mL sample was injected into a pre-calibrated Picarro G2201-i analyzer for analysis. Calibration standards from Air Liquide (Std 1: CO_2 concentration: 2050 µmol/mol, δ^{13}-CO_2: -18‰, Std 2: CO_2 concentration: 350 µmol/mol, $\delta^{13}C$-CO_2: -15‰, and Std 3: CH_4 concentration: 4 µmol/mol, $\delta^{13}C$-CH_4: -50‰) were used for calibration of the Picarro analyzer. Routine checks of optical and cavity parameters were conducted to assess instrument performance. Precision of the instrument was checked using 5 min average deviation of atmospheric air. Deviation less than 10% for CH_4 concentration in duplicate samples further confirmed adequate performance of the instrument. However, as deviations in natural abundance isotopic values were high in samples with low CH_4 concentrations, we have refrained from reporting $\delta^{13}C$-CH_4 values of *in situ* CH_4 profiles. Concentrations of dissolved CH_4 were calculated from the gas solubility of CH_4, based on the equilibrium water temperature and atmospheric pressure (Hamilton 2006).

The possible change in [CH_4] due to sample storage was evaluated by analyzing freshly collected samples on a LI-COR trace gas analyzer (LI-7810) during summer 2024. We observed a very high agreement (R^2 = 0.99) between measurements from LI-COR and Picarro instruments, indicating negligible effect of sample storage on CH_4 analyses.

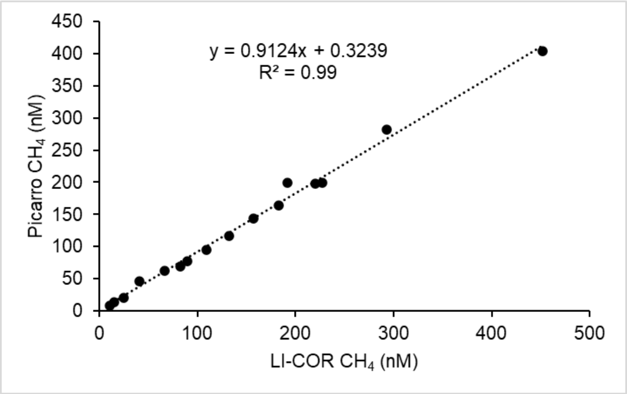

In situ dissolved inorganic carbon (DIC) concentrations were measured to calculate $^{13}C\text{-}CH_4$ production rates from $^{13}C\text{-}HCO_3$ in incubation experiments. Samples for DIC concentration analysis were acidified with HCl (Miyajima et al. 1995) prior to extraction by headspace equilibrium method (Roberts and Shiller 2015). Briefly, duplicate samples were acidified with 0.5 mL of 8 M HCl and thoroughly mixed. Subsequently, 50 mL of the acidified sample was transferred from the glass serum bottles into a 140 mL plastic syringe, and a 50 mL headspace was created with synthetic air. After vigorous shaking for 1 minute, the headspace was diluted at 1:10 with synthetic air and injected into a Picarro G2201-i analyzer and measured against a standard series of known concentrations of Na_2CO_3.

Text S2. Nutrient and Chlorophyll analysis

Dissolved inorganic nutrients were measured on a Turner Trilogy spectrofluorometer (Turner Designs, CA, USA) using established fluorometric and colorimetric methods, and quantified using external calibration curves of standards of known concentrations. Ammonium (NH_4^+) was measured fluorometrically following derivatization with o-phthaldialdehyde (OPA) (Holmes et al. 1999). Nitrite (NO_2^-) was measured colorimetrically using the Griess assay (García-Robledo et al. 2014), and nitrate (NO_3^-) was quantified using the vanadium(III) chloride reduction method

coupled to the Griess assay (García-Robledo et al. 2014). NO_3^- concentrations were determined by correcting for the absorbance of NO_2^-.

Owing to the oligotrophic nature of lakes Mondsee and Attersee, phosphate (PO_4^{3-}) concentrations were determined after pre-concentration using the MAGnesium Induced Co-precipitation (MAGIC) method (Anagnostou and Sherrell 2008) Briefly, $MgCl_2$ and NaOH were added to the sample to induce formation of brucite ($Mg(OH)_2$) for co-precipitation of PO_4^{3-}. The brucite pellets were recovered by centrifugation and dissolved in HCl prior to analysis using the molybdenum blue method (Murphy and Riley 1962). Potentially owing to phosphorus contamination associated with $MgCl_2$ and sample handling, the effective detection limit in this study (~250 nM) was lower than previously reported for the MAGIC method (0.15 nM, Anagnostou and Sherrell 2008). Chlorophyll *a* (Chl *a*) was measured after filtration onto 0.7 µm pore-size borosilicate glass microfiber filters. Pigments were subsequently extracted in 90% acetone under dark, cold conditions as described previously (Welschmeyer 1994). The instrument was calibrated with pure Chl *a* standards, using the non-acidification method to minimize interference from phaeopigments.

Quality assurance and quality control procedures included analysis of procedural blanks and calibration with freshly prepared standards. Instrument calibration curves exhibited linearity over the concentration ranges measured correlation coefficient (R^2) > 0.99. Method detection limits were determined as three times the standard deviation of blanks.

Text S3. DNA Extraction and 16S rRNA gene amplicon sequencing

DNA was extracted using the Qiagen PowerSoil Pro kit according to the manufactures' manual, with the following modifications for the processing of filters. After addition of CD1 buffer, samples were processed in a bead beater (40 s at 6 m/s), the supernatant was collected after

centrifugation (1 min, 15,000 *g*), 25µL of proteinase K (>600mUA mL^{-1}, Qiagen) was added, and samples incubated at 56°C for 4h. After cooling down of the samples, DNA extraction proceeded following the manufacturer's manual.

The V4 hypervariable region of the bacterial and archaeal 16S rRNA gene was amplified and sequenced on a Illumina MiSeq (2 x 300bp) sequencer following established protocols (Pjevac et al. 2021). Amplicon pools were extracted from the raw sequencing data using the FASTQ workflow in BaseSpace (Illumina) with default parameters. Raw data processing was performed as described previously (Pjevac et al. 2021). Demultiplexing was performed with the python package demultiplex (Laros JFJ, github.com/jfjlaros/demultiplex) allowing one mismatch for barcodes and two mismatches for linkers and primers. Amplicon sequence variants (ASVs) were inferred using the DADA2 R package v1.42 (Callahan et al. 2016a) applying the recommended workflow (Callahan et al. 2016b). FASTQ reads 1 and 2 were trimmed at 220 nt and 150 nt with allowed expected errors of 2. ASV sequences were subsequently classified using DADA2 and the SILVA database SSU Ref NR 99 release 138.2 (Callahan 2024).

Table S1. Environmental conditions and incubation conditions for ^{13}C-bicarbonate stable isotope tracing experiments to measure primary production-associated ^{13}C-CH_4 production.

Lake	Season	Sampling depth (m)	*In situ* temp (°C)	Incubation temp (°C)	Incubation light intensity (µmol photons m^{-2} s^{-1})	Incubation light quality*	Incubation time (h)	Light dark cycle (h/h)
Mondsee	summer 2024	7.5	17.5	17-18	30-50	White light	T1 = 12<br>T2 = 24	12/12
Mondsee	summer 2024	15	8.5	8-10	10	Deep blue light	T1 = 12<br>T2 = 24	12/12
Attersee	summer 2024	10	16.7	17-18	30-50	White light	T1 = 12<br>T2 = 24	12/12
Attersee	summer 2024	25	6.7	5-9	10	Deep blue light	T1 = 12<br>T2 = 24	12/12
Mondsee	autumn 2024	4	12.5	12.5	95-150	White light	T1 = 36<br>T2 = 72	8/16
Mondsee	autumn 2024	10	12.5	13.3	20-40	White light with steel blue filter	T1 = 36<br>T2 = 72	8/16
Attersee	autumn 2024	10	13.3	13.3	20-40	White light with steel blue filter	T1 = 48<br>T2 = 96	8/16
Attersee	autumn 2024	20	8.5	8.0-8.5	10	Deep blue light	T1 = 48<br>T2 = 96	8/16

*Light intensity and quality were adjusted using a combination of incubator white light, steel blue filters (LEE Filters, Toronto, Canada) and deep blue aquarium LED light (SolarStinger SunStrip, Econlux, Cologne, Germany).

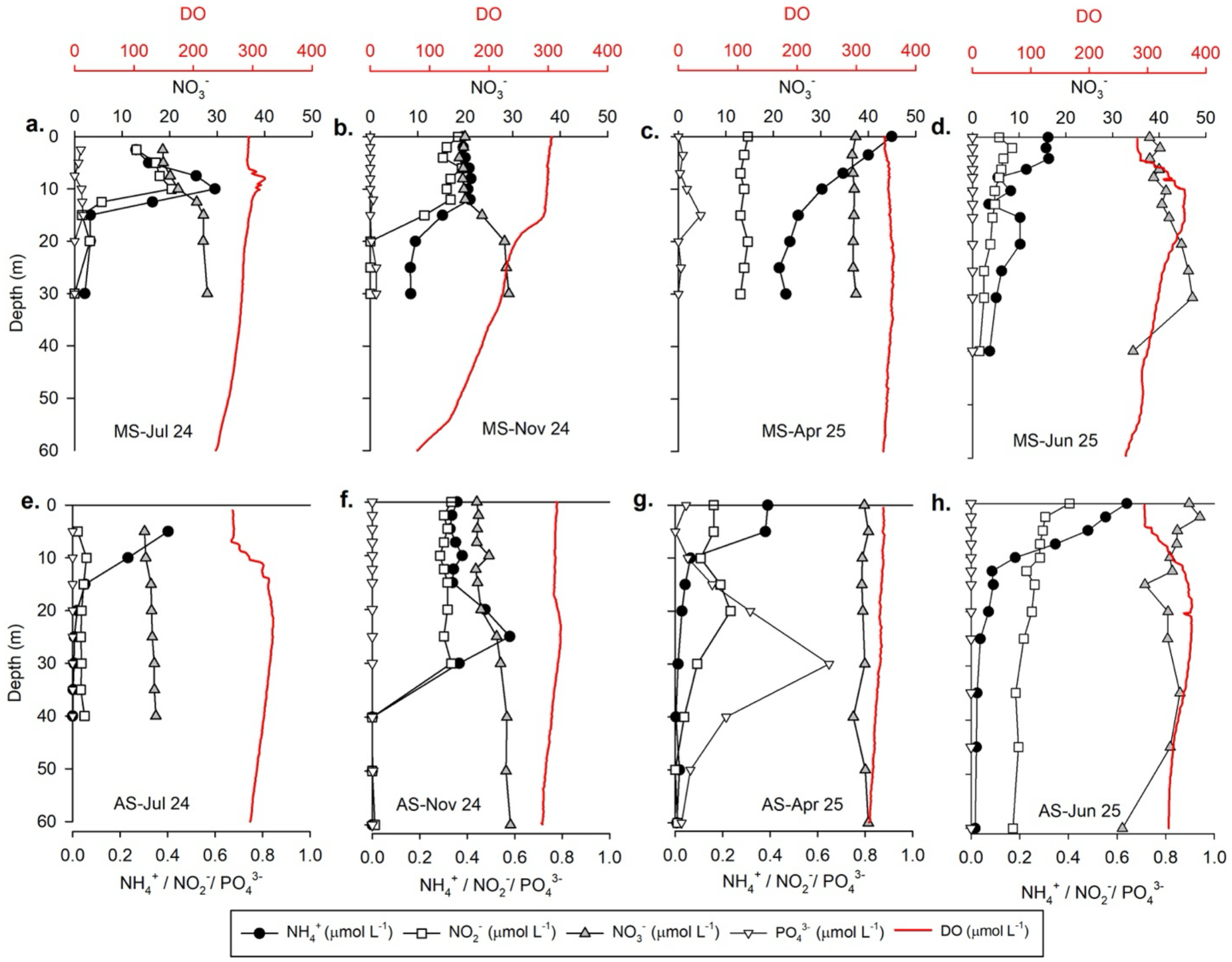

Figure S1. Vertical profiles of ammonium (NH_4^+), nitrite (NO_2^-), nitrate (NO_3^-), phosphate (PO_4^{3-}) and dissolved oxygen (DO) concentrations in lakes Mondsee (MS, a-d) and Attersee (AS, e-h) during different seasons. Sampling time corresponding to each plot is denoted by month-year. Full depth oxygen profiles (down to 150 m depth) for Attersee are available in Sharma et al. 2026.

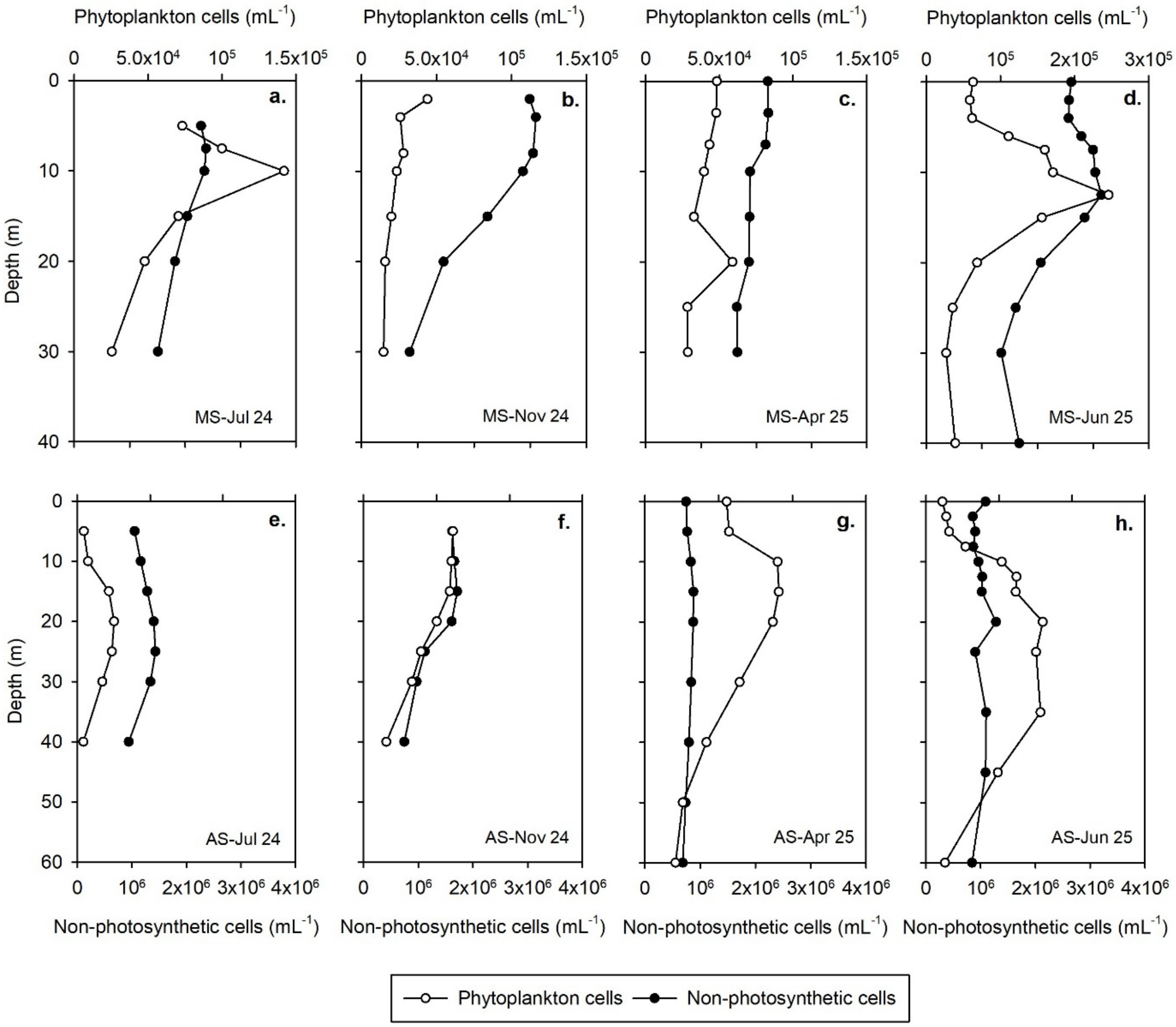

Figure S2. Vertical profiles of phytoplankton and non-photosynthetic cells in lakes Mondsee (MS, a-d) and Attersee (AS, e-h) during different seasons. Sampling time corresponding to each plot is denoted by month-year.

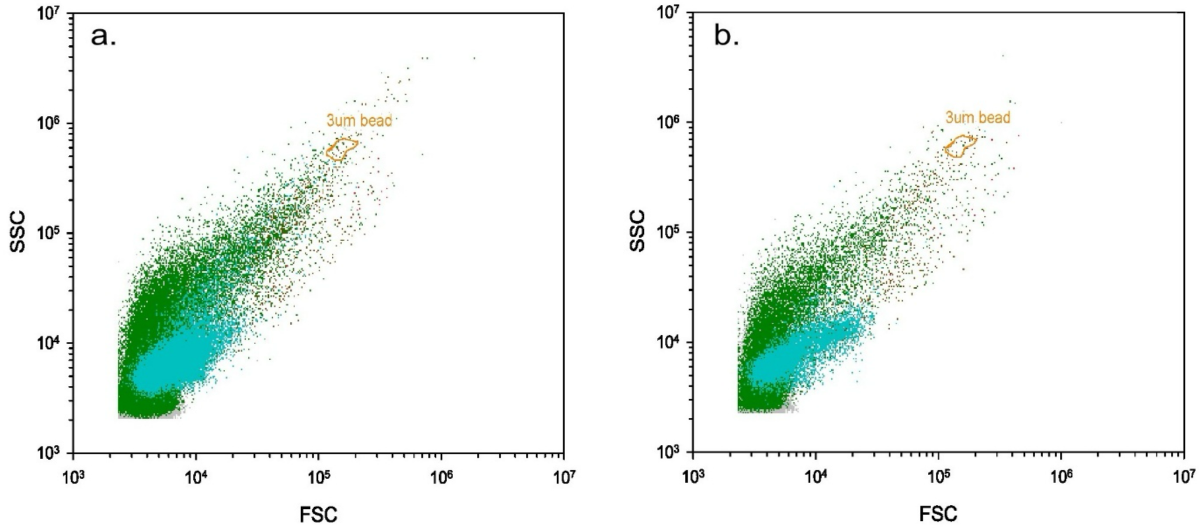

Figure S3. Size comparison of phytoplankton cells and 3 µm standard beads in lakes (a) Mondsee and (b) Attersee. The presented data shows samples collected from 12.5m depth and represents a typical flow cytometry side scatter (SSC) versus forward scatter (FSC) dot plot. Green dots represent SYBR Green-positive events (all cells) and cyan dots indicate events of cells containing chlorophyll and/or phycoerythrin (phytoplankton).

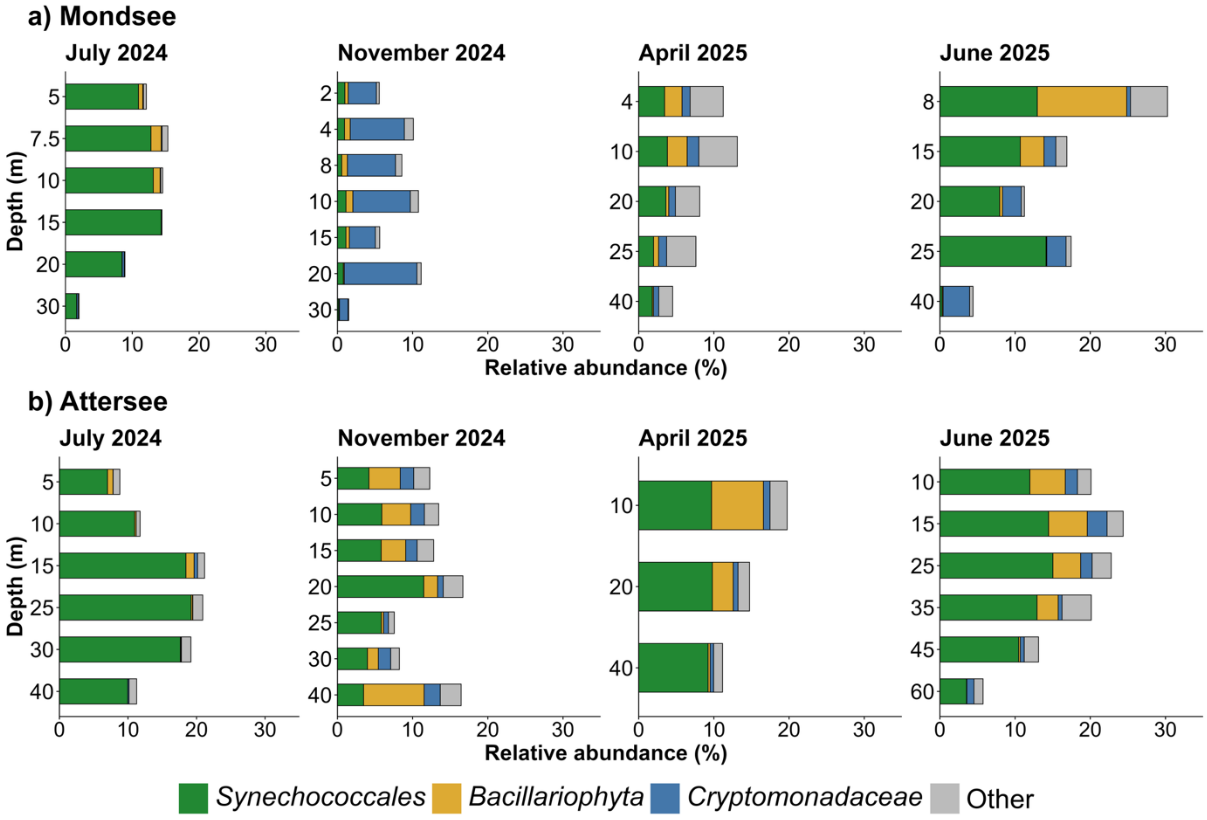

Figure S4. Phytoplankton community composition from 0.2-3 µm size fractions in lakes (a) Mondsee and (b) Attersee during different seasons. Shown are relative 16S rRNA gene amplicon sequence abundances of different phylogenetic groups. Phytoplankton groups which could not be further identified are summarized in the category "Other". Eukaryotic phytoplankton were identified by their chloroplast sequences.

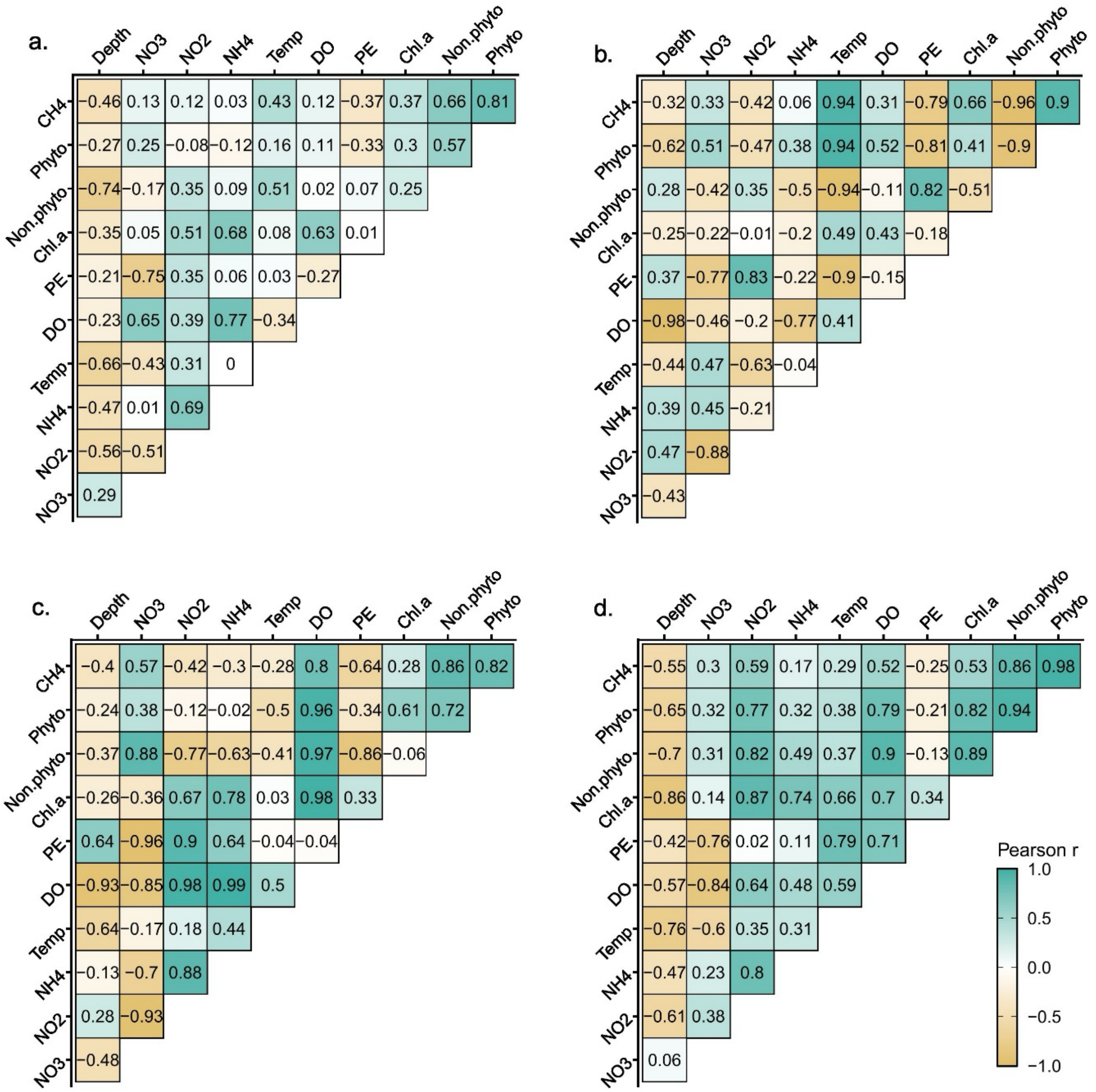

Figure S5. Pearson correlation matrices from correlation analyses between multiple environmental parameters in Mondsee for (a) all seasons and depths (n = 39), (b) epilimnion depths (n = 11), (c) metalimnion depths (n = 7), and (d) hypolimnion depths (n = 13). Correlation analysis for lake layers includes samples from the seasons where a distinct metalimnion was identified. Variables include: CH_4 = dissolved methane, Phyto = phytoplankton cells, Non-phyto = non-photosynthetic cells, Temp = temperature, DO = dissolved oxygen, Chl.a = chlorophyll a, PE = phycoerythrin, NH_4 = ammonium, NO_2 = nitrite, NO_3 = nitrate.

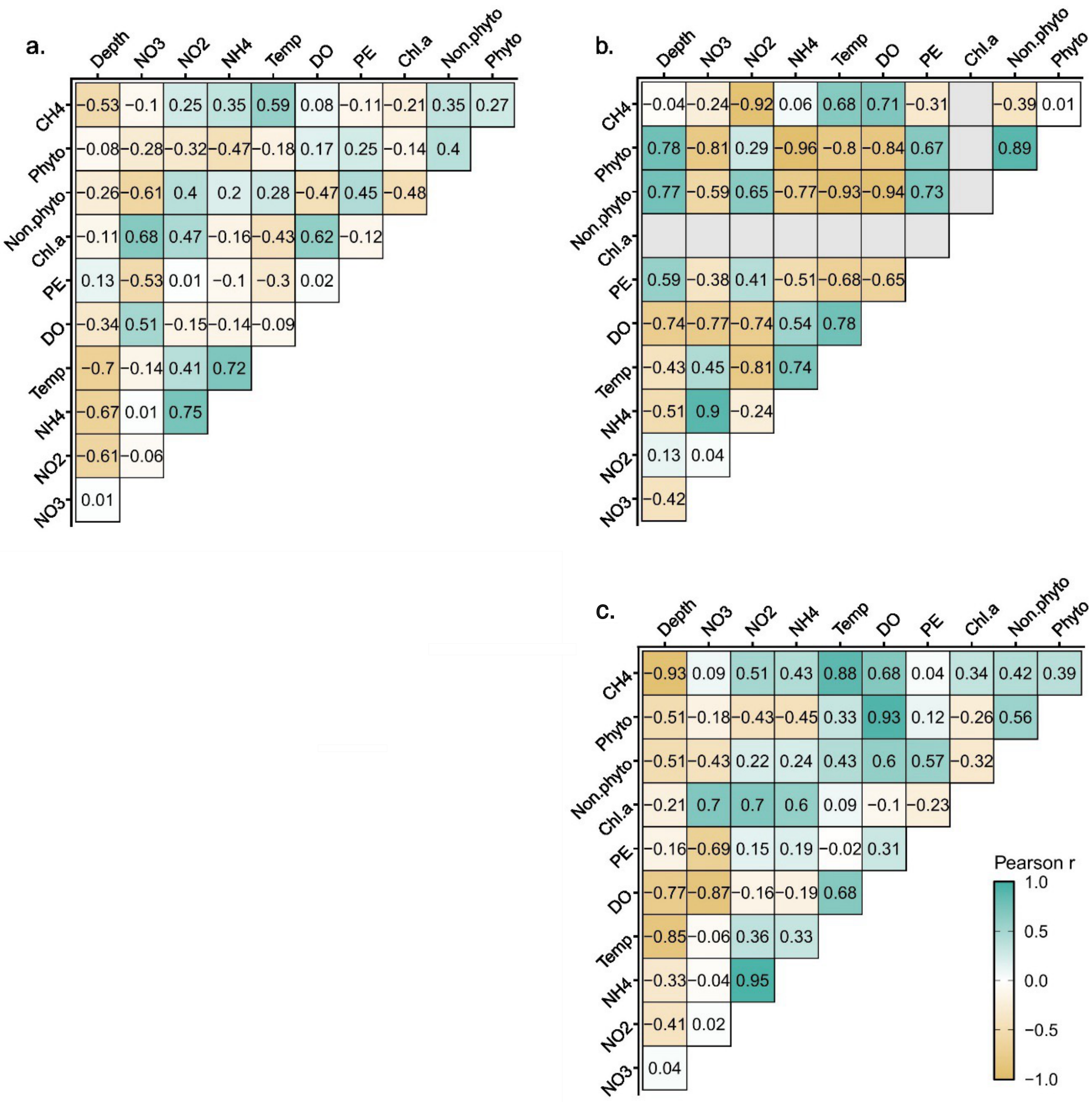

Figure S6. Pearson correlation matrices from correlation analyses between multiple environmental parameters in Attersee for (a) all seasons and depths (n = 42), (b) epilimnion depths (n = 10), (c) hypolimnion depths (n = 19). Correlation analysis for lake layers includes samples from the seasons where a distinct metalimnion was identified. Parameters with insufficient data to calculate correlations are indicated by grey tiles. Note that correlation analysis of metalimnion depths were excluded due to insufficient data (n=4). Variables include: CH_4 = dissolved methane, Phyto = phytoplankton cells, Non-phyto = non-photosynthetic cells, Temp = temperature, DO = dissolved oxygen, Chl.a = chlorophyll a, PE = phycoerythrin, NH_4 = ammonium, NO_2 = nitrite, NO_3 = nitrate.

References

Anagnostou, E., and R. M. Sherrell. 2008. MAGIC method for subnanomolar orthophosphate determination in freshwater. Limnol. Oceanogr. Methods **6**: 64–74. doi:10.4319/lom.2008.6.64

Callahan, B. 2024. Silva taxonomic training data formatted for DADA2 (Silva version 138.2).doi:10.5281/zenodo.14169026

Callahan, B. J., P. J. McMurdie, M. J. Rosen, A. W. Han, A. J. A. Johnson, and S. P. Holmes. 2016a. DADA2: High-resolution sample inference from Illumina amplicon data. Nat. Methods **13**: 581–583. doi:10.1038/nmeth.3869

Callahan, B., K. Sankaran, J. Fukuyama, P. McMurdie, and S. Holmes. 2016b. Bioconductor Workflow for Microbiome Data Analysis: from raw reads to community analyses [version 2; peer review: 3 approved]. F1000Research **5**. doi:10.12688/f1000research.8986.2

García-Robledo, E., A. Corzo, and S. Papaspyrou. 2014. A fast and direct spectrophotometric method for the sequential determination of nitrate and nitrite at low concentrations in small volumes. Mar. Chem. **162**: 30–36. doi:10.1016/j.marchem.2014.03.002

Hamilton, S. 2006. Calculation of dissolved gas concentrations and isotope ratios from measurements made after static headspace extraction.

Holmes, R. M., A. Aminot, R. Kérouel, B. A. Hooker, and B. J. Peterson. 1999. A simple and precise method for measuring ammonium in marine and freshwater ecosystems. Can. J. Fish. Aquat. Sci. **56**: 1801–1808. doi:10.1139/f99-128

Miyajima, T., Y. Miyajima, Y. T. Hanba, K. Yoshii, T. Koitabashi, and E. Wada. 1995. Determining the stable isotope ratio of total dissolved inorganic carbon in lake water by GC/C/IIRMS. Limnol. Oceanogr. **40**: 994–1000. doi:10.4319/lo.1995.40.5.0994

Murphy, J., and J. P. Riley. 1962. A modified single solution method for the determination of phosphate in natural waters. Anal. Chim. Acta **27**: 31–36. doi:10.1016/S0003-2670(00)88444-5

Pjevac, P., B. Hausmann, J. Schwarz, G. Kohl, C. W. Herbold, A. Loy, and D. Berry. 2021. An Economical and Flexible Dual Barcoding, Two-Step PCR Approach for Highly Multiplexed Amplicon Sequencing. Front. Microbiol. **12**: 669776. doi:10.3389/fmicb.2021.669776

Roberts, H. M., and A. M. Shiller. 2015. Determination of dissolved methane in natural waters using headspace analysis with cavity ring-down spectroscopy. Anal. Chim. Acta **856**: 68–73. doi:10.1016/j.aca.2014.10.058

Sharma, N., B. Bayer, and M. Felsberger. 2026. Limnological parameters related to seasonal differences and potential biological drivers of the methane paradox in Lake Attersee and Mondsee, Austria. Version v3 [Data set]. Zenodo. https://doi.org/10.5281/zenodo.19394022

Welschmeyer, N. A. 1994. Fluorometric analysis of chlorophyll a in the presence of chlorophyll b and pheopigments. Limnol. Oceanogr. **39**: 1985–1992. doi:10.4319/lo.1994.39.8.1985
